# Thoracic Endovascular Aortic Repair in Delayed Thoracic Aortic Pseudoaneurysm Caused by a Thoracic Vertebral Fracture

**DOI:** 10.70352/scrj.cr.25-0326

**Published:** 2025-09-03

**Authors:** Daiki Mori, Yusuke Ozaki, Dai Fukushima, Kohei Yamao, Ryosuke Usui, Hiroshi Fukuma, Shota Nakao, Tetsuya Matsuoka

**Affiliations:** Rinku General Medical Center, Senshu Trauma and Critical Care Center, Izumisano, Osaka, Japan

**Keywords:** delayed aortic injury, delayed aortic pseudoaneurysm, thoracic endovascular aortic repair, traumatic vertebral fracture

## Abstract

**INTRODUCTION:**

Thoracic vertebral fractures are commonly associated with aortic injury at the time of injury. However, delayed thoracic aortic injury during intensive care management of thoracic fractures has not been reported.

**CASE PRESENTATION:**

An 83-year-old man was transported to our hospital after a collision with a car while riding a motorized bicycle. A fracture of the 10th thoracic vertebra was identified and treated with thoracic fusion on day 24. On day 34, a new pseudoaneurysm was found in contact with the vertebral fracture. Thoracic endovascular aortic repair (TEVAR) was performed on day 36, and disappearance of the pseudoaneurysm was confirmed on day 38.

**CONCLUSIONS:**

The possibility of delayed thoracic aortic pseudoaneurysm should be considered when thoracic vertebral fractures are near the descending aorta. Early TEVAR may be effective for delayed aortic injury.

## Abbreviation

TEVAR
: thoracic endovascular aortic repair

## INTRODUCTION

Traumatic thoracic vertebral fractures have sometimes occurred with spinal cord injury and chylothorax,^1)^ and several cases of associated blunt thoracic aortic injury have been reported.^2,3)^ However, delayed thoracic aortic injury during intensive care management of thoracic vertebral fractures has not been documented. Here, we report a case of thoracic endovascular aortic repair (TEVAR) for a delayed thoracic aortic pseudoaneurysm secondary to a thoracic vertebral fracture.

## CASE PRESENTATION

An 83-year-old Japanese man was transported to our hospital after a traffic collision while riding a motorbike. His medical history included type 2 diabetes mellitus, hypertension, and dyslipidemia. On arrival, his vital signs were as follows: respiratory rate of 45/min; oxygen saturation, 95% on a reservoir mask at 15 L/min; heart rate, 156/min; peripheral coldness; blood pressure, 140/85 mmHg; Glasgow Coma Scale score, 14 (E4V4M6); and body temperature, 36.0°C. Physical examination revealed a laceration on the left mandible and contusions on the left clavicle, back of the left hand, right forearm, right upper arm, and both lower legs. Blood tests revealed anemia, hyperfibrinolysis, and elevated lactate levels (**Table 1**).

**Table 1:** Laboratory data on admission

Biochemistry			Blood cell count			Arterial blood gas analysis (F_I_O_2_ 0.4, PEEP 8cmH_2_O)		
CPK	380	U/L	WBC	18500	/uL	pH	7.384	
LD	269	U/L	Hb	12.3	g/dL	P/F	220	
AST	47	U/L	Plt	20.5	10^4^/uL	PO_2_	88	mmHg
ALT	41	U/L				PCO_2_	39.8	mmHg
ALP	63	U/L	Coagulation			HCO_3_^−^	23.2	mmol/L
γ-GTP	20	U/L	PT-INR	0.98		BE	−1.1	mmol/L
T-Bill	0.6	mg/dL	APTT	34.5	Second	Lactate	4.1	mmol/L
BUN	17.5	mg/dL	Fib	292	mg/dL			
Cr	0.89	mg/dL	D-dimer	59.1	ug/mL			
Sodium	136	mEq/L	FDP	172.6	ug/mL			
Potassium	4	mEq/L						
CRP	0.30	mg/dL						

ALP, alkaline phosphatase; ALT, alanine aminotransferase; APTT, activated partialthromboplastin time; AST, aspartate aminotransferase; BE, base excess; BUN, blood urea nitrogen; CPK, creatine phosphokinase; Cr, creatinine; CRP, C-reactive protein; FDP, fibrin degradation products; Fib, fibrinogen; γ-GTP, γ-glutamyltransferase; Hb, hemoglobin; LD, lactate dehydrogenase, P/F, PO_2_/F_I_O_2_ ratio; Plt, platelet; PT-INR, prothrombin time-international normalized ratio; T-Bill, total bilirubin; WBC, white blood cell.

**Fig. 1:**
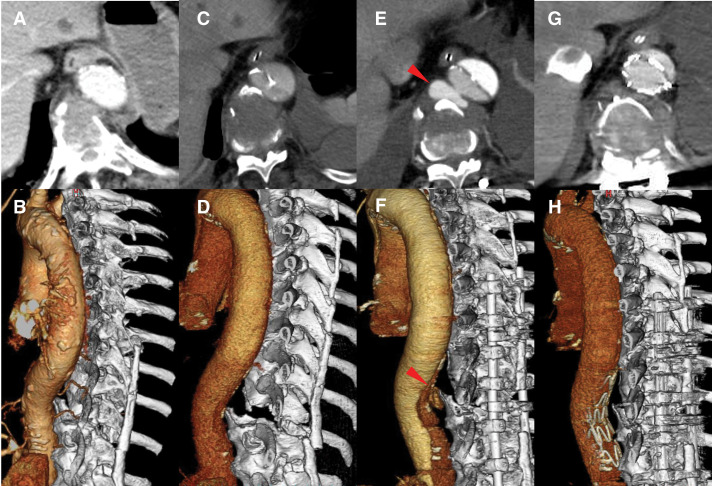
Time course of a delayed thoracic aortic pseudoaneurysm associated with a thoracic vertebral fracture. (**A**) and (**B**) CT reveals the 10th thoracic vertebra near the descending aorta on admission. (**C**) and (**D**) CT on day 19 reveals the 10th thoracic vertebra to be in contact with the descending aorta; however, no pseudoaneurysm is observed. (**E**) and (**F**) CT shows a new pseudoaneurysm in contact with the 10th thoracic vertebra on day 34 (arrows). (**G**) and (**H**) CT on day 38 confirms resolution of the pseudoaneurysm.

The patient was intubated because of an unstable respiratory status and circulation. CT revealed a cerebral contusion, traumatic subarachnoid hemorrhage, Stanford type A traumatic aortic dissection confined to the ascending aorta, and fractures of the left distal clavicle, left 2nd–8th ribs, right 3rd rib, 10th thoracic vertebra (AO Spine Thoracolumbar Injury Classification System type C), transverse process of the 2nd lumbar vertebra, mandibular, left distal ulna, and left 3rd and 4th metacarpals (Injury Severity Score: 29, probability of survival: 0.82). Aortic injury was not observed at the point of contact with the 10th thoracic vertebra (**Fig. 1A** and **1B**).

An intracranial pressure sensor for traumatic brain injury was placed for monitoring, and a negative pressure fixation device was fitted for the burst fracture of the 10th thoracic vertebral. Intensive care management began after suturing of the left mandible and left hand and external fixation of the left forearm.

Targeted temperature management for traumatic brain injury was provided from days 1 to 4. The patient was initially treated conservatively with antihypertensive treatment because of the presence of the traumatic subarachnoid hemorrhage and because the traumatic aortic dissection did not cause cardiac tamponade or mal-perfusion. CT on day 10 revealed aortic dissection extending from the ascending aorta to above the superior mesenteric artery; however, CT on day 19 (**Fig. 1C** and **1D**) revealed findings similar to those on day 10, with no pseudoaneurysm.

Thoracic spinal fusion was performed on day 24. On day 34, CT revealed a new pseudoaneurysm at the edge of the thoracic vertebral fracture (**Fig. 1E** and **1F**), and TEVAR was performed on day 36. CT on day 38 confirmed the resolution of the pseudoaneurysm (**Fig. 1G** and **1H**). CT on days 43 and 51 confirmed that the pseudoaneurysm had not recurred. The patient was moved from the ICU to the general ward on day 63 and transferred to the hospital on day 141.

## DISCUSSION

We showed that delayed thoracic aortic pseudoaneurysm was caused by thoracic vertebral fracture during intensive care management and that TEVAR was beneficial in its treatment.

First, we established that thoracic aortic injury can occur later as a comorbidity in patients with thoracic vertebral fractures. In previous reports, aortic injury was observed at the time of injury;^2,3)^ however, there have been no reports of cases where delayed aortic pseudoaneurysms have developed. Delayed aortic injury following traumatic rib fractures has been documented on day 4, which is thought to have resulted from ongoing mechanical irritation caused by the fractured rib.^4)^ In our case, the thoracic vertebral fracture of AO classification type C was highly unstable. We believe that the delayed thoracic aortic pseudoaneurysm resulted from continuous irritation caused by body movement and repositioning, consistent with the findings of a previous report.^4)^ Since aortic growth typically occurs within the 1st week after aortic dissection,^5)^ it is unlikely that the pseudoaneurysm developed in association with the dissection. However, as the aortic dissection extended to the descending aorta, the fragile aortic wall may have come into contact with the fractured thoracic vertebra, which could have increased the likelihood of pseudoaneurysm formation compared with that in non-dissected segments of the aorta. As delayed aortic injury caused by pedicle screws typically occurs months to years postoperatively,^6)^ chronic irritation from the vertebral fracture is more likely the cause than device-related factors. Therefore, a pseudoaneurysm observed on day 34 (10 days postoperatively) suggests that intraoperative positional changes and mechanical irritation during the procedure may have contributed to its development. In one case, a delayed aortic injury due to rib fractures was treated 3–11 days after injury^4)^; performing a CT with reference to a similar time period may, therefore, be reasonable for cases of delayed aortic injury due to vertebral fractures. Notably, delayed thoracic aortic pseudoaneurysms can occur in highly unstable thoracic vertebral fractures. Therefore, a CT scan should be performed when significant physical movement or positional changes occur.

Second, we demonstrated that TEVAR was effective for the treatment of delayed thoracic aortic injuries. Treatments for blunt thoracic aortic injury include open repair and TEVAR. Recently, the efficacy of TEVAR for blunt thoracic aortic injury has gained increasing recognition.^7)^ In our case, a delayed pseudoaneurysm secondary to a vertebral fracture was treated with TEVAR, suggesting its potential utility in the treatment of delayed aortic injury. Lagios et al. also reported on the effectiveness of TEVAR in treating delayed iatrogenic thoracic aortic pseudoaneurysm.^8)^

Preceding TEVAR has been shown to allow safe vertebral fracture surgery, even without existing aortic damage.^9)^ However, endoleaks are known complications of TEVAR,^10)^ and the risk of delayed aortic injury due to vertebral instability persists if vertebral fixation is not performed. Therefore, spinal fixation should be considered after prophylactic TEVAR to prevent such complications. TEVAR is less invasive, has a shorter operation time,^2,7)^ and can be performed in patients with poor general condition. Moreover, it can be performed in the supine position, eliminating the need for repositioning. These findings suggest that aortic injury can be prevented by performing TEVAR before vertebral fusion, thus potentially mitigating delayed pseudoaneurysm formation.

## CONCLUSIONS

Our case demonstrates that a delayed aortic pseudoaneurysm caused by a thoracic vertebral fracture can be effectively treated with TEVAR. For highly unstable vertebral fractures near the descending aorta, early consideration of TEVAR and close monitoring via CT should be considered, as delayed pseudoaneurysm may occur.

## DECLARATIONS

### Availability of data and materials

The data that support the findings of this study are available from the corresponding author upon reasonable request.
